# Exploring the relationship between dispositional mindfulness and hoarding behavior: A moderated multi-mediation model

**DOI:** 10.3389/fpsyg.2022.935897

**Published:** 2022-08-12

**Authors:** Yanping Gong, Yuxuan Tan, Rong Huang

**Affiliations:** School of Business, Central South University, Changsha, China

**Keywords:** mindfulness, hoarding, self-esteem, emotion dysregulation, gender differences

## Abstract

Hoarding behavior may not only interfere with hoarders’ daily lives but may also endanger the community. However, few studies have investigated the role of personality characteristics in hoarding behavior. We hypothesized that dispositional mindfulness would be negatively associated with hoarding behavior, and tested mechanisms and gender differences in this association. An online survey was conducted in a sample of 533 Chinese adults (262 women, *M*_age_ = 26.82; *SD* = 6.30). Regression-based analyses showed that mindfulness was associated with less hoarding behavior through higher self-esteem and lower emotion dysregulation. Moreover, gender moderated the mediating effect of emotion dysregulation in the association between mindfulness and hoarding behavior. Specifically, the indirect association was only significant for women. These findings provide a deeper understanding of how, why, and for whom dispositional mindfulness is negatively associated with hoarding behavior, they provide support for self-completion theory and the cognitive-behavioral model of hoarding, and they have heuristic value for future research.

## Introduction

Hoarding behavior refers to acquiring but failing to use or discard numerous seemingly useless objects, what has been called “pathological collecting” (Frost and Gross, 1993; Frost et al., 2009). Hoarding behavior is relatively common in the population, with about 2.3∼5.8% of people showing significant problems (Samuels et al., 2008; Turna et al., 2018; Liu et al., 2021). Hoarding can interfere with daily life in the form of compulsive buying (Frost et al., 2011), social fears (Samuels et al., 2002), and depression symptoms (Raines et al., 2016). It may also endanger the surrounding community by increasing the risk of fires caused by clutter, injuries from falls, and the spread of bacteria (e.g., McGuire et al., 2013; Orr et al., 2019). Therefore, it is necessary to explore the antecedents of hoarding behavior, and develop effective methods to manage it.

Several studies have identified factors that may increase the risk of hoarding. For instance, anthropomorphism was found to be positively related to hoarding behavior (Burgess et al., 2018). Attachment anxiety was also positively associated with hoarding behavior *via* intolerance of uncertainty and experiential avoidance (Jia et al., 2019). Meanwhile, several studies have focused on the inhibitors of hoarding. Besides social support (Medard and Kellett, 2014) and social trust (Weiss et al., 2020), the attention facet of mindfulness skills has also been found to be negatively associated with compulsive hoarding symptoms for men who were on quarantine during the pandemic (Marazziti et al., 2021).

Mindfulness refers to an awareness and focus on the moment-to-moment experience in an open, non-reactive, and non-judgmental manner (Kabat-Zinn, 2003). Mindfulness can be enhanced through various interventions, such as meditation and mindfulness training (Kabat-Zinn, 2003). However, mindfulness can also be conceptualized as a trait or disposition. Dispositional mindfulness is an individual’s ability and tendency to maintain a state of mindfulness over time (Brown and Ryan, 2003; Brown et al., 2007). It has been suggested that almost all people have the ability to be mindful, but there are still individual differences in dispositional mindfulness (Pepping and Duvenage, 2016). Individuals with higher dispositional mindfulness have a clear awareness of what is actually happening in the moment, and therefore make conscious decisions and show less reflexive and impulsive behavior (Brown and Ryan, 2003). Although a recent study found the attention facet of mindfulness skills was negatively associated with compulsive hoarding symptoms (Marazziti et al., 2021), the relationship between individual differences in dispositional mindfulness and hoarding behavior, as well as the mechanisms underlying this relationship, have not yet been tested.

Moreover, many studies have always focused on gender differences in the mindfulness effects (e.g., Rojiani et al., 2017; Brem et al., 2019; Kingery et al., 2021). A neuroscience study demonstrated that men and women may benefit from different components of meditation to achieve similar effects (Luders et al., 2015). Other research also noted that different genders may benefit from mindfulness through various processes and at different levels (Katz and Toner, 2013). It is important to determine the effect of mindfulness on hoarding behavior by gender and how it works among men and women in our study. Thus, our study tested whether, why, and for whom dispositional mindfulness and hoarding behavior might be negatively correlated.

## Theoretical framework and hypotheses

### Mindfulness and hoarding behavior

Hoarding is characterized by excessive acquisition and difficulty in discarding large numbers of seemingly useless objects (Greenberg et al., 1990; Frost and Gross, 1993). Previous research found that mindfulness was positively associated with self-control (e.g., Bowlin and Baer, 2012; Canby et al., 2015; Li et al., 2021), which was negatively linked with the tendency to save items, as well as difficulty in discarding them (Carbonella and Timpano, 2016).

Moreover, individuals with higher mindfulness focus not only on their internal processes but also on their surroundings (Shapiro et al., 2006). They live in the present moment so that they are not overwhelmed and guided by thoughts, emotions, and feelings in their actions and choices (Shapiro et al., 2006). Individuals with higher mindfulness may be more likely to make reasonable and non-emotional decisions (Shapiro et al., 2012), and therefore may be less likely to engage in hoarding behaviors.

Recent investigations have included mindfulness as a component of an intervention for obsessive-compulsive symptoms (e.g., Patel et al., 2007; Sugiura and Sugiura, 2015; Apaolaza et al., 2019), and one of the symptom dimensions is hoarding disorder. Moreover, previous research found that individuals with compulsive hoarding symptoms scored lower in the attention facet of mindfulness skills than those without such symptoms (Marazziti et al., 2021). These studies jointly suggest the negative association between mindfulness and hoarding behavior.

### The mediating role of self-esteem

Self-esteem was expected to act as a mediator of the association between mindfulness and hoarding behavior in the current study. Self-esteem involves a positive or negative orientation toward the self (Rosenberg, 1965), reflecting a sense of self-acceptance and self-worth. Several studies have demonstrated a correlation between mindfulness and higher self-esteem (e.g., Park and Dhandra, 2017; Dhandra, 2020; Bharti et al., 2022). Mindfulness-based interventions have also been shown to significantly improve self-esteem (e.g., Ree and Craigie, 2007; Goldin et al., 2009). Mindfulness enables individuals to show non-judgment, make choices that are non-reactive, and be open to their experiences (Baer et al., 2006). In turn, mindful individuals are thought to be less judgmental and critical of themselves (Hildebrandt et al., 2017). Moreover, individuals with higher mindfulness are able to pay attention to current experiences rather than negative self-related thoughts, and let go of negative cognitions (Frewen et al., 2008). Thus, it is reasonable to propose a positive relationship between mindfulness and self-esteem.

Individuals with low self-esteem maintain a negative self-concept (Bharti et al., 2022). According to self-completion theory (Wicklund and Gollwitzer, 1982), individuals with negative self-concepts may seek ways to build their self-worth (Banister and Hogg, 2004). Possessions are perceived to be extensions of the self and significant others (Claes et al., 2016; Kings et al., 2021), which has been found to be positively correlated with hoarding symptoms and beliefs (Kings et al., 2021). Hoarders may believe that owning objects is more important than using them, and discarding these objects makes them feel like they have lost part of their identity (Foster et al., 2007). That is, individuals may engage in hoarding behaviors to get a sense of self-identity and establish their self-worth (Kings et al., 2017, 2021). Thus, it is reasonable to infer the negative relationship between self-esteem and hoarding behavior.

Together, self-completion theory (Wicklund and Gollwitzer, 1982), along with these studies, suggests that mindfulness may be negatively associated with hoarding behavior *via* an increase in self-esteem. Specifically, individuals with higher mindfulness may be able to reduce their judgmental and critical attitudes toward themselves and therefore show higher self-esteem (e.g., Park and Dhandra, 2017; Dhandra, 2020; Bharti et al., 2022), with higher self-esteem, they become less likely to build their self-worth by hoarding.

### The mediating role of emotion dysregulation

Emotion dysregulation may act as another mediator of the association between mindfulness and hoarding behavior. Emotion dysregulation involves an impaired ability to experience, express, and use emotions (Eisenberg et al., 2000). Previous research showed a correlation between mindfulness and lower emotion dysregulation (e.g., Luberto et al., 2014; Cheung et al., 2020; Powers et al., 2021). Moreover, after 6 weeks of training focused meditation (FM), healthy college students reported significantly lower scores in emotion dysregulation (Menezes and Bizarro, 2015). As mindfulness involves being open and accepting emotions, rather than ruminating or avoiding these experiences (Shapiro et al., 2006), individuals with higher mindfulness may have lower emotion dysregulation.

Emotions have been shown to be important in understanding hoarding. Hoarding is characterized by emotionally reinforced saving behaviors, as hoarders generally have strong emotional attachments to numerous objects (e.g., Mathes et al., 2020; Yap and Grisham, 2021). The cognitive-behavioral model of hoarding (Frost and Hartl, 1996) suggests that emotions play a significant role in hoarding onset and maintenance. Specifically, negative emotions are associated with avoidance (e.g., refusal to discard), whereas positive emotions are associated with approach behaviors (e.g., acquisition) (Frost and Hartl, 1996; Mathes et al., 2020). To avoid the negative emotions associated with discarding, hoarders will refuse to discard objects, and therefore saving is reinforced and maintained (Taylor et al., 2018). Meanwhile, to keep the positive emotions associated with acquiring new possessions and finding valuable possessions through clutter, hoarders may engage in more acquisitions and savings (e.g., Frost and Hartl, 1996; Steketee and Frost, 2003; Taylor et al., 2018).

As hoarders often acquire items to regulate both positive and negative emotions (Tolin, 2011), and difficulties in emotion regulation have been found to be associated with more hoarding symptoms (e.g., Fernandez de la Cruz et al., 2013; Taylor et al., 2018; Tolin et al., 2018). The cognitive-behavioral model of hoarding (Frost and Hartl, 1996), along with these studies, suggests that individuals with higher levels of mindfulness may have less emotion dysregulation, which in turn may reduce the likelihood that they will engage in hoarding behaviors.

### The sequential mediating role of self-esteem and emotion dysregulation

The relationship between mindfulness and hoarding behavior may be serially mediated by self-esteem and emotion dysregulation. Individuals with higher mindfulness are less judgmental and critical of themselves, and therefore show higher self-esteem (e.g., Park and Dhandra, 2017; Dhandra, 2020; Bharti et al., 2022). Moreover, individuals with higher self-esteem could be better able to cope with stress (Perlin et al., 1981), and to experience less distress and depression (Rosenberg et al., 1989). The negative association between self-esteem and emotion dysregulation has been established in previous empirical studies (e.g., Garofalo et al., 2016; Zhang et al., 2017). Low self-esteem involves negative self-evaluation beliefs (Smith et al., 2006); strong negative emotional reactions to unfavorable self-evaluations may in turn disrupt individuals’ ability to regulate emotions (Zeigler-Hill et al., 2014). Thus, hoarding (e.g., saving possessions) may be an attempt to regulate emotions related to identity issues (e.g., Frost and Hartl, 1996; Morrison and Johnson, 2011; Tolin, 2011). This logic, along with previous findings, suggests the possibility of a serial mediation effect linking mindfulness and hoarding behavior.

In brief, individuals with higher mindfulness show higher self-esteem (e.g., Park and Dhandra, 2017; Dhandra, 2020; Bharti et al., 2022), with higher self-esteem, they may have lower emotion dysregulation (e.g., Garofalo et al., 2016; Zhang et al., 2017) and ultimately, less hoarding behaviors (e.g., Fernandez de la Cruz et al., 2013; Taylor et al., 2018; Tolin et al., 2018).

### Gender differences: The moderating role of gender

It is possible that the mediation processes of interest differ for men and women. One consideration is that there appear to be gender differences in each of the key variables in our study. Table 1 lists studies that have found gender differences in mindfulness, self-esteem, emotion dysregulation, and hoarding. Each entry includes information about the sample and outcomes. With few exceptions, men had higher scores for mindfulness (e.g., Dhandra and Park, 2018; Amemiya and Sakairi, 2020; Webb et al., 2021) and self-esteem (e.g., Bleidorn et al., 2016; Rentzsch et al., 2016; Magee and Upenieks, 2019), and women had higher scores for emotion dysregulation (e.g., Barrett et al., 2000; Lafrance Robinson et al., 2014; Wieckowski et al., 2020) and hoarding (e.g., Neave et al., 2016; Tripathi et al., 2018; Turna et al., 2018).

**TABLE 1:** Summary of studies reviewed in the gender differences for mindfulness, self-esteem, emotion dysregulation, and hoarding.

Constructs	Author, year	Sample details	Main findings
Mindfulness	Amemiya and Sakairi, 2020	*N* = 152 (25 women; *M*_age_ = 19.52, *SD*_age_ = 1.89), university athletes in Japan	Female athletes had lower dispositional mindfulness than male athletes.
	Dhandra and Park, 2018	*N* = 146 (50% women) aged 18–26, university students in India	Men scored higher on the mindfulness scale than women.
	Gilbert and Waltz, 2010	*N* = 269 (185 women; *M*_age_ = 20.9), volunteers from a mid-sized Western university	Women were significantly higher in the Observe factor, and men were significantly higher on the non-react factor.
	Webb et al., 2021	*N* = 201 (63% women; *M*_age_ = 13.24), eighth grade students across nine Baltimore City public middle schools	Males reported significantly greater levels of trait mindfulness than their female peers.
	Josefsson et al., 2011	*N* = 92, including 45 university students (31 women; *M*_age_ = 26, *SD*_age_ = 6.3) recruited from Halmstad University’s School of Social and Health Science, and 47 meditators (26 women; *M*_age_ = 44, *SD*_age_ = 11.3) recruited from eight Swedish Buddhist centers and two non-religious meditation groups in Halmstad	In the total sample, men had significantly higher scores than women on the non-react scale. In the experienced meditation group, men scored higher than women on the non-react scale. Among non-meditators, women scored higher than men on the Observation scale.
Self-esteem	Magee and Upenieks, 2019	*N* = 4005 (2215 women) aged 30–84 in wave-2; *N* = 2699 (1,488 women) aged 39–93 in wave-3, a United States national sample	Observed a baseline gender difference in self-esteem, with men scoring slightly higher than women.
	Bleidorn et al., 2016	*N* = 985,937 (60% women) aged 16–45, a large internet sample across 48 nations (Argentina, Australia, Austria, Belgium, Bolivia etc.)	Men consistently reporting higher self-esteem than women.
	Rentzsch et al., 2016	*N* = 1009 (541 women) aged 13–65, adolescent and adult in Germany	Female participants showed lower social, academic, and physical self-esteem as well as lower self-regard than male participants.
	Orth et al., 2010	*N* = 3,617 (62% women) aged 25–104, a national four-wave panel survey of individuals who live in the contiguous United States	Women had lower self-esteem than did men in young adulthood, but their trajectories converged in old age.
	McMullin and Cairney, 2004	*N* = 16,051 (8890 women) aged 12 and older, Canadian residents across its 10 provinces	In all age groups, women had lower levels of self-esteem than men.
Emotion Dysregulation	Wieckowski et al., 2020	*N* = 722 (146 women) aged 4–20, an ASD inpatient psychiatric sample	Female psychiatric inpatients had more severe dysregulation, including higher reactivity and dysphoria, than inpatient males.
	Šeibokaitė et al., 2017	*N* = 246 (129 women) aged 19–75, non-professional Lithuanian drivers	Women scored significantly higher on the non-acceptance of Emotional Responses scale than men, but men reported greater emotional awareness problems than women.
	Lafrance Robinson et al., 2014	*N* = 250 (165 women) aged 17–50, a non-clinical sample of men and women from undergraduate university programs	Women reported greater difficulties with a number of emotion regulation skills, including emotional clarity, the ability to engage in goal-directed behavior, and the ability to use adaptive strategies to regulate an emotion.
	Gratz and Roemer, 2004	*N* = 357 (260 women) aged 18–55, students from undergraduate psychology courses offered at the University of Massachusetts Boston	Men reported greater emotional awareness problems than women.
	Barrett et al., 2000	*N* = 1217 (total of seven samples)	Women scored higher on a performance test of emotional awareness than did men.
Hoarding	Tripathi et al., 2018	*N* = 945 (381 women) aged 18–72, diagnosis of obsessive-compulsive disorder (OCD) from 16 participating centers (in the northern part of India)	Women had a higher prevalence of hoarding compulsions than men (15.5% vs. 9.8%).
	Turna et al., 2018	*N* = 729 (66.7% women) aged 16–77, users of online classified advertisements	Individuals in the High Hoarding Rating Scale group were more likely to be women than men.
	Neave et al., 2016	*N* = 283 (210 women) aged 18–62, an opportunity sample	Women showed more hoarding tendencies than men.
	Steketee et al., 2015	*N* = 443 (304 women) aged 18–83, clinical (OCD) and community control samples	Participants reported more hoarding among female (mothers, sisters) than male (fathers, brothers) relatives.
	Samuels et al., 2008	*N* = 742 (465 women) aged 34–94, adult household residents of east Baltimore	The prevalence of hoarding was over two times as great in men (5.6%) compared with women (2.6%).
	Wheaton et al., 2008	*N* = 473 (289 women; *M*_age_ = 38.62; *SD*_age_ = 13.01), OCD patients from the adult OCD Clinic at the National Institute of Mental Health	Hoarding phenotype differed across genders, with female hoarders experiencing more severe OCD symptoms, earlier age of OCD onset, and a broader range of psychiatric comorbidity.

In tests of mediation, the question is whether these gender differences affect the strength or direction of one or more of the paths in the model, constituting moderated mediation. In the current study, we tested whether gender moderated the first path of each mediated pathway. First, gender may moderate the effect of mindfulness on self-esteem. This focus is justified because gender differences have been identified both in regard to self-esteem (e.g., Bleidorn et al., 2016; Magee and Upenieks, 2019) and in regard to the effect of mindfulness on self-compassion (e.g., Rojiani et al., 2017; Kang et al., 2018). As a complimentary self-concept to self-esteem, self-compassion refers to being accepting and caring of oneself (Kabat-Zinn, 2003; Neff, 2003; Hwang et al., 2016), and individuals with higher self-compassion generally show higher self-esteem (Neff, 2011). The positive effect of mindfulness on self-compassion has been shown to be stronger in women than in men (e.g., Rojiani et al., 2017; Kang et al., 2018). These findings suggest that the effect of mindfulness on self-esteem may be stronger in women than in men. Moreover, individuals with higher self-esteem become less likely to build their self-worth by hoarding, which may in turn make the negative relationship between mindfulness and hoarding behavior different for men and women.

Second, gender may moderate the effect of mindfulness on the other mediator in our model, namely emotion dysregulation. The evidence here is based on the assumption that rumination in response to negative moods can be considered a form of emotion dysregulation (Zawadzki, 2015). Rumination has been found to have stronger relationships with psychological health, namely depression, than other emotion regulation strategies (Zawadzki, 2015). Previous research found that women ruminate more in response to depressive moods, thus exacerbating the moods (Nolen-Hoeksema et al., 1999). Moreover, mindfulness was found in general to be associated with less rumination, and the association was stronger in women than in men (Kingery et al., 2021). Thus, it is reasonable to infer that the effect of mindfulness on decreasing emotion dysregulation may be stronger in women than in men. Furthermore, as for the positive correlation between emotion dysregulation and hoarding (e.g., Fernandez de la Cruz et al., 2013; Taylor et al., 2018; Tolin et al., 2018), the gender difference of mindfulness in decreasing emotion dysregulation may then spill over into hoarding behavior.

### The present study

**FIGURE 1:**
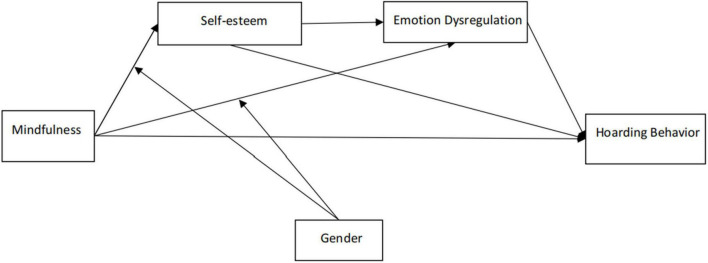
Overview of the proposed moderated multi-mediation model.

Guided by self-completion theory (Wicklund and Gollwitzer, 1982) and the cognitive-behavioral model of hoarding (Frost and Hartl, 1996), we administered online questionnaires to a community sample of adults to test two parallel mediation processes (self-esteem as the mediator; emotion dysregulation as the mediator) and a sequential mediation process (self-esteem and emotion dysregulation as sequential mediators). Gender was tested as a moderator of the mediating pathways. Together, these tests constitute a test of a moderated mediation model. See Figure 1. We made the following hypotheses:

Hypothesis 1. Mindfulness will be negatively associated with hoarding behavior.

Hypothesis 2. Mindfulness will be negatively associated with hoarding behavior *via* an increase in self-esteem.

Hypothesis 3. Mindfulness will be negatively associated with hoarding behavior *via* a decrease in emotion dysregulation.

Hypothesis 4. Mindfulness will be negatively associated with hoarding behavior *via* a sequence of increased self-esteem and decreased emotion dysregulation.

Hypothesis 5. Gender will moderate the mediating effect of self-esteem in the association between mindfulness and lower hoarding behavior. Specifically, the indirect association will be stronger in women than in men.

Hypothesis 6. Gender will moderate the mediating effect of emotion dysregulation in the association between mindfulness and lower hoarding behavior. Specifically, the indirect association will be stronger in women than in men.

## Materials and methods

### Participants

The study employed a cross-sectional design. The inclusion criteria were the age of 18 years old or older. To ensure the validity of our data, we remove the questionnaires where (a) duplicate IP addresses, (b) incorrect responses to the screening items, (c) answering the questions in less than 120 s, (d) marks of answers of items show regular shapes (such as zigzag patterns) (e.g., Wills et al., 2008; Bao et al., 2015, 2016). There were 533 Chinese adults (262 women, *M*_age_ = 26.82; *SD* = 6.30) who provided valid reports, resulting in a valid response rate of about 70%. These participants came from 29 provinces or municipalities in China, with the smallest number from Ningxia Province (*N* = 1, 0.19% of the total sample) and the largest number from Guangdong Province (*N* = 66, 12.38% of the total sample). Table 2 provides descriptive statistics, demographic information, and gender differences on the study variables. As shown in Table 2, most participants were pursuing or had a bachelor’s degree (59.47%). Their incomes were diverse, with 28.89% earning a monthly income from 5,001 to 8,000 RMB (about 792–1,266 USD), followed by 27.95% with a monthly income from 2,000 to 5,000 RMB (about 317–792 USD). There was no gender difference on age (*t* (531) = 0.50, *p* > 0.05), mindfulness (*t* (531) = 0.45, *p* > 0.05), self-esteem (*t* (531) = 1.23, *p* > 0.05), emotion dysregulation (*t* (517) = −1.79, *p* > 0.05), or hoarding behavior (*t* (498) = −1.95, *p* > 0.05). Women had a higher education level than men (*Z* = −3.34, *p* < 0.001), and men had a higher income level than women (*Z* = −2.98, *p* < 0.01).

**TABLE 2:** Demographic information and results of the comparisons between genders.

Variables	Whole sample (*N* = 533)	Men (*N* = 271)	Women (*N* = 262)	Difference between groups
	
	*N* (%)	*N* (%)	*N* (%)	
Age (years, range 18–55)	*M* = 26.82, *SD* = 6.30	*M* = 26.69, *SD* = 6.13	*M* = 26.96, *SD* = 6.48	*t* (531) = 0.50
Education level				
High school diploma	36 (6.75)	22 (8.12)	14 (5.34)	*Z* = −3.34***
Junior college degree	95 (17.82)	54 (19.93)	41 (15.65)	
Bachelor degree	317 (59.47)	167 (61.62)	150 (57.25)	
Master degree and above	85 (15.95)	28 (10.33)	57 (21.76)	
Monthly income (Unit: Yuan RMB)				
<2,000	88 (16.51)	34 (12.55)	54 (20.61)	*Z* = −2.98**
2,000–5,000	149 (27.95)	69 (25.46)	80 (30.53)	
5,001–8,000	154 (28.89)	87 (32.10)	67 (25.57)	
8,001–10,000	96 (18.01)	56 (20.66)	40 (15.27)	
>10,000	46 (8.63)	25 (9.23)	21 (8.02)	
CAMS-R	*M* = 2.80, *SD* = 0.36	*M* = 2.79, *SD* = 0.33	*M* = 2.80, *SD* = 0.39	*t* (531) = 0.45
RSES	*M* = 4.33, *SD* = 0.90	*M* = 4.29, *SD* = 0.87	*M* = 4.37, *SD* = 0.94	*t* (531) = 1.23
DERS	*M* = 2.90, *SD* = 0.83	*M* = 2.96, *SD* = 0.77	*M* = 2.83, *SD* = 0.88	*t* (517) = −1.79
SI-R	*M* = 1.75, *SD* = 0.71	*M* = 1.80, *SD* = 0.62	*M* = 1.69, *SD* = 0.78	*t* (498) = −1.95

CAMS-R, Cognitive and Affective Mindfulness Scale-Revised; RSES, Rosenberg Self-Esteem Scale; DERS, Difficulties in Emotion Regulation Scale; SI-R, Saving Inventory-Revised.

**p < 0.01, ***p < 0.001.

### Procedure

The study was approved by the research ethics committee at the corresponding author’s institution. We collected online data for adults in China from October 1 to 3, 2021. Specifically, we produced and distributed the questionnaire through the online survey tool Questionnaire Star. Potential participants were informed about the study through advertisements on the authors’ WeChat Moments and a professional questionnaire collection agency in China. Moreover, the participants were told that when they finished the questionnaires they could, if they wished, forward the questionnaire link to others. When participants clicked on the survey hyperlink, they saw an information sheet that clearly explained the purpose and procedures of the study, the fact that participation was voluntary and they had the right to withdraw at any time, and that no personally identifiable information was collected. Participants provided their informed consent before completing the questionnaires. Each participant was given a small payment for participating after the investigation to thank them for their time and effort.

### Measures

All the instruments we used in our research were well-developed scales used in previously published studies. We used the translation and back-translation method to translate these scales into Chinese.

#### Dispositional mindfulness

Dispositional mindfulness was assessed with the Cognitive and Affective Mindfulness Scale-Revised (CAMS-R) (Feldman et al., 2007). The scale includes 12 items on four dimensions: attention, present focus, awareness, and acceptance. A sample item is “I am able to focus on the present moment.” The 12 items are rated on a four-point Likert scale (1 = rarely/not at all; 4 = almost always). After reverse-coding of negatively worded items, responses to all items were averaged, with higher scores indicating higher mindfulness. The Cronbach’s α of mindfulness for this sample was 0.72. After controlling the reverse wording effect, the results of confirmatory factor analysis (CFA) showed acceptable structural validity: χ^2^/*df* = 2.64; CFI = 0.94; TLI = 0.92; IFI = 0.94; RMSEA = 0.06.

#### Self-esteem

Self-esteem was assessed with the Rosenberg Self-Esteem Scale (RSES) (Rosenberg, 1965), a ten-item unidimensional scale. A sample is “I take a positive attitude toward myself.” One reversed item “I wish I could have more respect for myself” was excluded for cultural differences (Tian, 2006). The nine remaining items are rated on a six-point Likert scale (1 = strongly disagree; 6 = strongly agree). After reverse-coding of negatively worded items, responses to all items were averaged, with higher scores indicating higher self-esteem. The Cronbach’s α of self-esteem for this sample was 0.91. After controlling the reverse wording effect, the results of CFA showed acceptable structural validity: χ^2^/*df* = 3.56; CFI = 0.98; TLI = 0.97; IFI = 0.98; RMSEA = 0.07.

#### Emotion dysregulation

Emotion dysregulation in the current study was measured with the Difficulties in Emotion Regulation Scale (DERS) (Bjureberg et al., 2016). The scale includes 16 items and is structured by five dimensions: lack of emotional clarity, difficulties engaging in goal-directed behavior, impulse control difficulties, limited access to effective emotion regulation strategies, and non-acceptance of emotional responses. A sample is “When I am upset, I have difficulty controlling my behaviors.” The 16 items are rated on a five-point Likert scale (1 = almost never; 5 = almost always). A higher average score of 16 ratings reflected higher emotion dysregulation. The Cronbach’s α of emotion dysregulation for this sample was 0.96. CFA of the DERS showed acceptable structural validity: χ^2^/*df* = 2.98; CFI = 0.97; TLI = 0.96; IFI = 0.97; RMSEA = 0.06.

#### Hoarding behavior

Hoarding behavior was measured with the Saving Inventory-Revised scale (SI-R) (Frost et al., 2004). The scale includes 23 items and is structured by three dimensions: clutter, difficulty discarding, and acquisition. A sample is “To what extent do you have difficulty throwing things away?” The 23 items are rated on a five-point Likert scale (0 = not at all; 4 = very often/extreme). Responses to all items were averaged, with higher scores indicating more hoarding behaviors. The Cronbach’s α of hoarding behavior for this sample was 0.94. CFA of the SI-R showed acceptable structural validity: χ^2^/*df* = 3.07; CFI = 0.94; TLI = 0.93; IFI = 0.94; RMSEA = 0.06.

#### Control variables

Control variables were age, education level, and income level. Previous research showed that younger people exhibited stronger hoarding cognition and behaviors (Neave et al., 2015). Education level has been found to be positively and significantly related to self-esteem (de Araujo and Lagos, 2013). Education level has also been found to be related to hoarding, with the participants who have a college or professional school degree scoring significantly higher than the other participants (Danet, 2018). Moreover, income level has been found to be positively related to self-esteem and hoarding (Samuels et al., 2008; de Araujo and Lagos, 2013). Therefore, we selected age, education level, and income level as statistical control variables.

## Results

All the hypotheses were tested with the SPSS PROCESS macro (Hayes, 2013), a computational tool for testing moderation and mediation effects as well as their combinations. Models 6 and 84 of the SPSS PROCESS macro were employed to test the hypothesized model. Moreover, the bootstrapping method was conducted to test the indirect effects (5,000 bootstrap samples). Indirect effects are considered significant when their confidence interval does not include 0.

### Preliminary analysis

Pearson correlations among mindfulness, self-esteem, emotion dysregulation, and hoarding behavior are presented in Table 3 for men and women, respectively. There were significant correlations among all study variables.

**TABLE 3:** Results of pairwise correlation analysis of all variables.

Variables	1	2	3	4	5	6	7
1. Age	–	−0.21*	0.39***	0.26***	0.22**	−0.18**	−0.17**
2. Education level	−0.20**	–	0.03	−0.10	0.04	0.00	0.02
3. Income level	0.40***	0.15*	–	0.39***	0.23***	−0.19**	−0.12*
4. CAMS-R	0.09	0.11	0.14*	–	0.70***	−0.60***	−0.45***
5. RSES	0.07	0.19**	0.18**	0.63***	–	−0.75***	−0.62***
6. DERS	−0.05	−0.23***	−0.11	−0.39***	−0.46***	–	0.73***
7. SI-R	−0.06	−0.13*	−0.10	−0.32***	−0.43***	0.74***	–

Correlations for men and women are presented below and above the diagonal, respectively.

N = 533 (271 men; 262 Women).

CAMS-R, Cognitive and Affective Mindfulness Scale-Revised; RSES, Rosenberg Self-Esteem Scale; DERS, Difficulties in Emotion Regulation Scale; SI-R, Saving Inventory-Revised.

*p < 0.05, **p < 0.01, ***p < 0.001.

### Testing for the main effect and mediation effects

**FIGURE 2:**
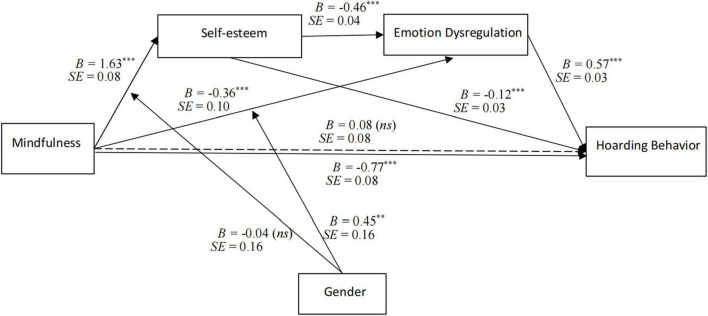
Regression results of the moderated multi-mediation model. The model is assessed using PROCESS macro (MODEL6 and MODEL84). Dotted line represents the direct effect of mindfulness on hoarding behavior; the effects of age, education level, and income level are controlled; ns, non-significant; **p < 0.01, ***p < 0.001.

The analysis results of SPSS PROCESS macro are presented in Figure 2 and Table 4. Mindfulness was negatively associated with hoarding behavior (*B* = −0.77, *p* < 0.001), supporting Hypothesis 1. Moreover, the total effect of mindfulness on hoarding behavior (effect = −0.77, 95% CI = [−0.93, −0.61]) was negative and significant, but the direct effect of mindfulness on hoarding behavior (effect = 0.08, 95% CI = [−0.08, 0.23]) was not significant.

**TABLE 4:** Regression results of MODEL6 and MODEL84.

Predictors	Equation 1 (SI-R)		Equation 2 (RSES)		Equation 3 (DERS)		Equation 4 (SI-R)	
				
	*B*	*SE*	*B*	*SE*	*B*	*SE*	*B*	*SE*
Age	−0.01	0.01	0.01	0.01	0.00	0.00	0.00	0.00
Education level	−0.05	0.04	0.11**	0.04	−0.06	0.04	0.03	0.03
Income level	0.02	0.03	0.00	0.03	0.02	0.03	0.01	0.02
CAMS-R	−0.77***	0.08	1.63***	0.08	−0.36***	0.10	0.08	0.08
RSES					−0.46***	0.04	−0.12***	0.03
DERS							0.57***	0.03
Gender			−0.05	0.06	0.06	0.06		
CAMS-R × gender			−0.04	0.16	0.45**	0.16		
*R* ^2^	0.16***		0.45***		0.42***		0.55***	

N = 533.

CAMS-R, Cognitive and Affective Mindfulness Scale-Revised; RSES, Rosenberg Self-Esteem Scale; DERS, Difficulties in Emotion Regulation Scale; SI-R, Saving Inventory-Revised.

**p < 0.01, ***p < 0.001.

Table 4 (Equations 2 and 4) shows that mindfulness was positively associated with self-esteem (*B* = 1.63, *p* < 0.001), and self-esteem was negatively associated with hoarding behavior (*B* = −0.12, *p* < 0.001). Moreover, Table 4 (Equations 3 and 4) shows that mindfulness was negatively associated with emotion dysregulation (*B* = −0.36, *p* < 0.001), and emotion dysregulation was positively associated with hoarding behavior (*B* = 0.57, *p* < 0.001). Meanwhile, Table 4 (Equations 3) shows that self-esteem was negatively associated with emotion dysregulation (*B* = −0.46, *p* < 0.001).

In addition, Table 5 shows that the simple and serial mediation effects of self-esteem and emotion dysregulation were statistically significant (mindfulness→self-esteem→hoarding behavior: effect = −0.20, 95% CI = [−0.33, −0.07]; mindfulness→emotion dysregulation→hoarding behavior: effect = −0.22, 95% CI = [−0.34, −0.10]; mindfulness→self-esteem→emotion dysregulation→hoarding behavior: effect = −0.43, 95% CI = [−0.55, −0.32]). Thus, two parallel mediation paths and the sequential mediation path of self-esteem and emotion dysregulation were identified, supporting Hypotheses 2, 3, and 4.

**TABLE 5:** Results of indirect effects (5,000 bootstraps).

Path	Effect	SE	95% LLCI	95% ULCI
Total indirect effect	−0.84	0.08	−1.01	−0.69
CAMS-R→RSES→SI-R	−0.20	0.07	−0.33	−0.07
CAMS-R→DERS→SI-R	−0.22	0.06	−0.34	−0.10
CAMS-R→RSES→DERS→SI-R	−0.43	0.06	−0.55	−0.32

N = 533.

CAMS-R, Cognitive and Affective Mindfulness Scale-Revised; RSES, Rosenberg Self-Esteem Scale; DERS, Difficulties in Emotion Regulation Scale; SI-R, Saving Inventory-Revised.

### Testing for the moderated mediation effect

Table 4 shows the results of tests of moderated mediation. Table 4 (Equation 2) shows that the interaction of mindfulness and gender was not significantly related to self-esteem (*B* = −0.04, *p* > 0.05). Hence, Hypothesis 5 was not supported.

**FIGURE 3:**
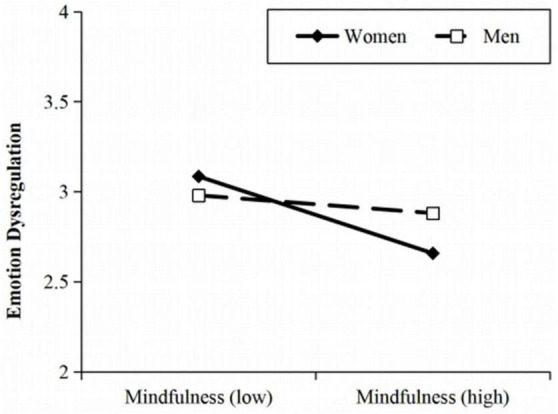
Moderating effect of gender on the relationship between mindfulness and emotion dysregulation.

As for Hypothesis 6, Table 4 (Equation 3) shows that the interaction of mindfulness and gender was positively associated with emotion dysregulation (*B* = 0.45, *p* < 0.01). Figure 3 illustrates the moderating effect of gender on the relationship between mindfulness and emotion dysregulation. Simple slopes tests revealed that the effect of mindfulness on emotion dysregulation was significant for women (*B*_simple_ = −0.59, *p* < 0.001, 95% CI = [−0.84, −0.35]), but not significant for men (*B*_simple_ = −0.14, *p* > 0.05, 95% CI = [−0.40, 0.13]). Meanwhile, the mediation effect of emotion dysregulation was significant for women (effect = −0.34, 95% CI = [−0.48, −0.19]), but not significant for men (effect = −0.08, 95% CI = [−0.24, 0.08]). Taken together, the results support Hypothesis 6.

## Discussion

Our results showed that individuals with higher dispositional mindfulness had reported less hoarding behaviors. Specifically, individuals with higher mindfulness had higher self-esteem, which in turn was associated with lower emotion dysregulation, and ultimately less hoarding. Moreover, tests of gender differences showed that the effect of mindfulness on hoarding behavior through decreased emotion dysregulation was only significant in women, but there was no gender difference in the effect of mindfulness on hoarding behavior through self-esteem.

Sociocultural factors might partly explain why gender did not moderate the mediating effect of self-esteem in our sample. A sociocultural perspective holds that gender differences in self-esteem are largely governed by social influences that vary by context and culture (Kling et al., 1999; Orth and Robins, 2014). In many Asian countries, such as China, there are small gender differences in self-esteem, as people are more likely to engage in within-gender social comparisons that reduce self-stereotyping processes (Guimond et al., 2007; Bleidorn et al., 2016). Chinese culture is described as more collectivistic, and gender differences in self-esteem have been found relatively less in this cultural context (Bleidorn et al., 2016). Moreover, Buddhism, a religion that has existed in China for thousands of years, advocates reacting mindfully to maladaptive feelings, and being kinder and more accepting of oneself (David et al., 2013). It appears that the philosophy of Buddhism, and particularly the emphasis placed on mindfulness (Kuan, 2008; Jones-Smith, 2012), may influence Chinese people in a subtle way. Recent research showed that there was no significant gender difference in the extent to which mindfulness was associated with self-esteem in a Chinese sample (Wang and Kong, 2019). These studies might help to explain why there was no gender difference in the positive association between mindfulness and self-esteem in our sample. Mindfulness may be an important source of self-esteem for both men and women.

### Theoretical implications

The theoretical implications of the current study’s results include three aspects. First, the finding that mindfulness was negatively associated with hoarding behavior is in accordance with another recent study. Marazziti et al. (2021) found that the ability to regulate attention can protect from hoarding and play the role of a psychological factor associated with lower hoarding. Unlike the socio-psychological antecedents of hoarding that have received a lot of attention from researchers (e.g., Wheaton et al., 2011; Jia et al., 2019), the current study enriches and extended prior literature on the role of dispositional mindfulness in hoarding behavior.

The second notable contribution of the present study is that it sheds light on why dispositional mindfulness could be negatively associated with hoarding behavior, employing self-completion theory (Wicklund and Gollwitzer, 1982) and the cognitive-behavioral model of hoarding (Frost and Hartl, 1996). The results demonstrated the parallel and serial mediation effects of self-esteem and emotion dysregulation in the relationship between mindfulness and hoarding behavior. These findings suggest that mindfulness can help achieve a “double dividend” in decreasing hoarding behavior. Although previous studies showed the potential negative influence of mindfulness on hoarding behavior (Marazziti et al., 2021), our findings identify the relationship between dispositional mindfulness and hoarding behavior from a new perspective, and reveal the “black-box” of how this relationship works.

Finally, the present study provides a better understanding of the association between mindfulness and hoarding behavior by revealing its gender differences. The relationship between mindfulness and hoarding behavior through decreased emotion dysregulation was only significant in women. This finding is a response to the call for more research on gender differences in the mindfulness effects (Luders et al., 2015), provides new support to previous studies showing that the mindfulness effect is stronger in women than in men (e.g., Rojiani et al., 2017; Kang et al., 2018), and enriches research on gender differences in mindfulness and hoarding (e.g., Turna et al., 2018; Amemiya and Sakairi, 2020).

### Practical implications

The results also have potential applied value. Our results suggest that living and behaving more mindfully may benefit individuals in having less hoarding behaviors. Promoting mindfulness broadly may reduce hoarding behavior in non-clinical samples. Given the limitations of the study, any therapeutic implications we can offer are tentative, we await further clinical replication before strong treatment recommendations can be made. Our findings suggest decreasing hoarding by fostering mindfulness may be a beneficial focus for future intervention. Randomized controlled trials would provide the strongest evidence of the effectiveness of mindfulness-based interventions to decrease hoarding. It is possible that this type of intervention could be effective not just in reducing hoarding, but also in influencing the contributing factors of self-esteem and low emotion dysregulation. Moreover, we found an important gender difference that can be taken into account in future interventions. Our findings showed that the indirect association between mindfulness and hoarding behavior *via* a decrease in emotion dysregulation was only significant in women. Interventions to reduce emotion dysregulation and hoarding could be tailored separately for men and women.

### Limitations and future directions

Our study has several limitations that can be addressed in future research. First, limited by the cross-sectional design, we cannot make causal inferences about the relations among mindfulness, self-esteem, emotion dysregulation, and hoarding behavior. In our research, prior experimental/longitudinal studies (e.g., Ree and Craigie, 2007; Goldin et al., 2009; Menezes and Bizarro, 2015; Cheung et al., 2020), the self-completion theory (Wicklund and Gollwitzer, 1982), and the cognitive-behavioral model of hoarding (Frost and Hartl, 1996) are helpful in conceptualizing possible causal links. Still, it is problematic to use a cross-sectional design to study mediation effects as estimates may be biased and may not be replicated in longitudinal research (Maxwell et al., 2011). Longitudinal or experimental designs should be conducted in the future to verify causality and demonstrate the predictive power of the mediation model.

Second, the present study was not conducted with a clinical sample, so the findings should be applied with caution to clinically hoarding adults. Although a community sample might provide useful information about hoarding in a natural context as hoarders do not necessarily come to the attention of mental health providers (Büscher et al., 2013), the study should be repeated in a clinical sample to assess the clinical generalizability of the relationships and to explore if more severely hoarding adults would benefit from mindfulness.

Third, we did not measure whether participants have attention problems, a factor that has been shown to influence hoarding (e.g., Hartl et al., 2005; Hallion et al., 2015). Neither did we measure participants’ levels of self-compassion, a factor that was related to participants’ mindfulness (e.g., Rojiani et al., 2017; Kang et al., 2018). Future research should measure these factors as control variables and rule out these factors as alternative explanations.

Fourth, we were interested in hoarding generally and therefore did not analyze the relationship between mindfulness and each aspect of hoarding (clutter, difficulty discarding, and acquisition). Although this total score has been often used (e.g., Neave et al., 2015, 2016), some studies suggest that it is more necessary to look at each aspect separately (e.g., Timpano et al., 2011; Ayers et al., 2014). It is possible that mindfulness is more strongly associated with compulsive acquisition than other aspects of hoarding, as the beneficial effects of mindfulness in reducing impulsive or compulsive buying have been well established (e.g., Park and Dhandra, 2017; Dhandra, 2020; Brunelle and Grossman, 2022). Individuals with higher levels of mindfulness are more satisfied with the possessions they already have, they have less interest in consumerist messages and aspirations for more (Brown et al., 2009). Thus, further investigation is needed to comprehensively understand how mindfulness relates to various aspects of hoarding and whether there are significant differences in the relationship between mindfulness and different aspects of hoarding.

Although not a limitation, it should be noted that the study was carried out in China and so the generalizability of the results needs to be tested. Buddhism is a part of Chinese culture, and its devotees emphasize mindfulness meditation (Xu et al., 2014). Moreover, the self-control associated with hoarding could be affected by the individualistic or collectivistic cultural orientation. Greater importance is placed on self-control in more collectivistic societies (e.g., China) than in individualistic societies (e.g., the United States) (Timpano et al., 2015). Thus, future research could consider studying the links between mindfulness and hoarding behavior in different cultural contexts.

## Conclusion

The current study examined “how” and “why” dispositional mindfulness and hoarding were negatively correlated. Through an online questionnaire survey to a community sample of 533 adults in China, we explored the effect of dispositional mindfulness on hoarding behavior, and tested mechanisms and gender differences in this association. Specifically, mindfulness was negatively associated with hoarding behavior through the parallel and sequential mediating pathways of increased self-esteem and decreased emotion dysregulation. Moreover, the indirect effect of mindfulness and hoarding behavior through decreased emotion dysregulation was only significant in women. The results of the study shed light on how self-esteem and emotion dysregulation are associated with the process of the mindfulness-hoarding relationship, contribute to the existing work on gender differences in mindfulness and hoarding behavior, and have heuristic value for future research.

## Data availability statement

The original contributions presented in this study are included in the article/supplementary material, further inquiries can be directed to the corresponding author.

## Ethics statement

The studies involving human participants were reviewed and approved by Central South University Institutional Review Board. The patients/participants provided their written informed consent to participate in this study.

## Author contributions

YG contributed to conception and design of the study, assisted with the execution of the study and data collection, and provided critical revisions. YT contributed to conception and design of the study, executed the study, analyzed the data, and drafted the manuscript. RH assisted with the data collection and drafted the manuscript. All authors contributed to manuscript reversion, read and approved the submitted version.
